# Takayasu arteritis complicated by SAPHO syndrome: A case-based review

**DOI:** 10.1007/s10067-025-07501-0

**Published:** 2025-05-22

**Authors:** Shu Sugimoto, Dai Kishida, Tatsuya Kobayashi, Naoki Tanomogi, Jun-Ichi Kurashina, Takanori Ichikawa, Yasuhiro Shimojima, Yoshiki Sekijima

**Affiliations:** Department of Medicine (Neurology & Rheumatology), Shinshu University School of Medicine, 3-1-1 Asahi, Matsumoto, 390-8621 Japan

**Keywords:** Takayasu arteritis, SAPHO syndrome, Chest pain, Biological agents

## Abstract

Takayasu arteritis (TAK) is often associated with other inflammatory diseases. Here, we describe two Japanese patients with TAK complicated by synovitis, acne, pustulosis, hyperostosis, and osteitis (SAPHO) syndrome. Both patients presented with anterior chest pain as their chief complaint and were diagnosed with TAK following SAPHO syndrome. Treatment with glucocorticoids and biological agents led to a rapid improvement in symptoms. A review of the literature identified 11 additional cases of TAK complicated by SAPHO syndrome. SAPHO syndrome frequently precedes TAK, with the diagnostic interval between the two diseases ranging from 1 month to 12 years. No clear association was found between the sites of osteoarticular and vascular involvement. These findings suggest that SAPHO syndrome may be a comorbid condition in patients with TAK. As TAK may develop several years after the diagnosis of SAPHO syndrome, clinicians should consider the possibility of TAK in patients presenting with severe inflammation that cannot be fully explained by SAPHO syndrome alone.

## Introduction

Synovitis, acne, pustulosis, hyperostosis, and osteitis (SAPHO) syndrome is a rare inflammatory osteoarticular disorder [1]. Although the diagnostic criteria established by Benhamou et al. [2] and Kahn et al. [3] are widely used, SAPHO syndrome is considered a spectrum of heterogeneous disorders characterized by osteoarticular manifestations with or without skin lesions. This concept encompasses several conditions, including chronic recurrent multifocal osteomyelitis (CRMO), acquired hyperostosis syndrome (AHS), and pustulotic arthro-osteitis (PAO) [4]. The anterior chest wall (ACW) is the most commonly affected site and a hallmark feature of SAPHO syndrome [5, 6].

Takayasu arteritis (TAK) is a chronic large-vessel vasculitis that primarily affects the aorta and its major branches, leading to arterial stenosis, occlusion, or dilatation [7]. The disease often presents with systemic symptoms such as fever, malaise, and fatigue, as well as head and neck symptoms, including dizziness and headache [8]. TAK has been associated with various inflammatory diseases, including inflammatory bowel disease [9], spondyloarthritis (SpA) [10], and pyoderma gangrenosum [11]. However, its coexistence with SAPHO syndrome is rare and not well understood.

Herein, we describe two patients with TAK who initially presented with anterior chest pain and were subsequently diagnosed with SAPHO syndrome. Additionally, we review similar cases reported in the literature to further characterize the clinical relationship between these two conditions.

## Case presentation

### Case 1

**Fig. 1:**
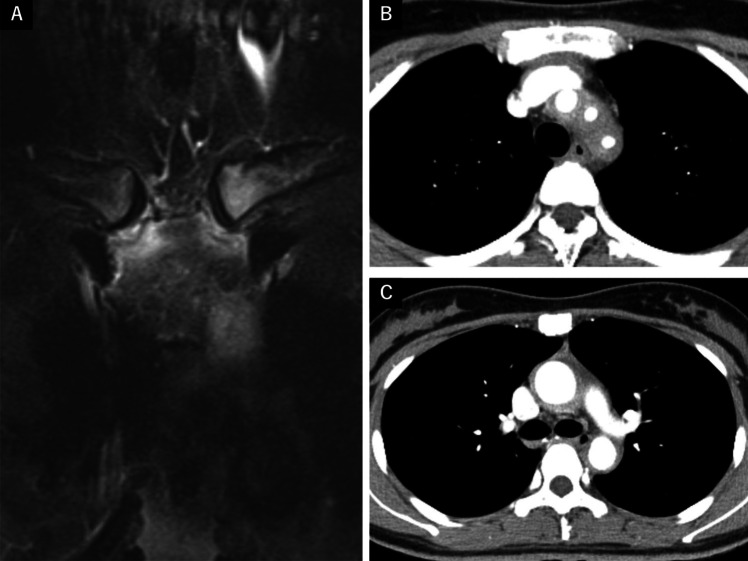
**A** Chest magnetic resonance imaging showing high-intensity areas in the sternoclavicular and sternocostal joints. **B C** Chest computed tomography demonstrating circumferential thickening with a contrast effect of the ascending to descending aorta, bilateral common carotid arteries, and the left subclavian artery

A 27-year-old woman was referred to our hospital with anterior chest pain. She had developed pustules on her palms and soles 7 years earlier and had been experiencing anterior chest pain for the past 5 years. She was initially suspected of having PAO; however, she continued to receive treatment with topical steroids from the dermatologist and NSAIDs from the physician. Biotin was added three years ago, but her symptoms did not improve, prompting a referral to a dermatologist at another hospital. Chest magnetic resonance imaging (MRI) was performed to evaluate the anterior chest pain, and she was subsequently referred to our department due to suspected inflammation around the sternoclavicular joint and aorta. Physical examination revealed a body temperature of 37.0 °C, and a bruit was auscultated on the left side of her neck. No difference in blood pressure was observed between her arms, and there was no claudication or numbness in either upper extremity. She exhibited swelling of the sternoclavicular and sternocostal joints, tenderness at multiple tendon attachment sites in the extremities, and pustules on her palms and soles. Laboratory examinations revealed an elevated C-reactive protein (CRP) level (3.8 mg/dL; normal, < 0.10 mg/dL), while antinuclear antibodies (ANA), rheumatoid factor (RF) and anti-cyclic citrullinated peptide antibodies (ACPA) were negative. Chest MRI showed high-intensity areas in the sternoclavicular and sternocostal joints (Fig. 1A), as well as thickened vessel walls in the aortic arch and its branches, including the descending aorta. Contrast-enhanced computed tomography (CT) confirmed arterial wall thickening, stenosis of the ascending and descending aorta, and narrowing of the left-dominant bilateral common carotid arteries and left subclavian arteries (Fig. 1B, C). No collaterals were observed. The patient was diagnosed with SAPHO syndrome based on Benhamou’s criteria and concurrent TAK based on the 2022 American College of Rheumatology/European League Against Rheumatism (ACR/EULAR) classification criteria [12]. The patient was treated with prednisolone (PSL) (50 mg/day) and infliximab (250 mg per infusion) initiated on day 9 of treatment. Although CRP levels normalized and joint symptoms improved, moderate liver dysfunction developed after 6 months. Infliximab was discontinued and replaced with ustekinumab, leading to stabilization of her condition. PSL was successfully tapered and discontinued 2.5 years after treatment initiation, and she remains stable on ustekinumab monotherapy.

### Case 2

**Fig. 2:**
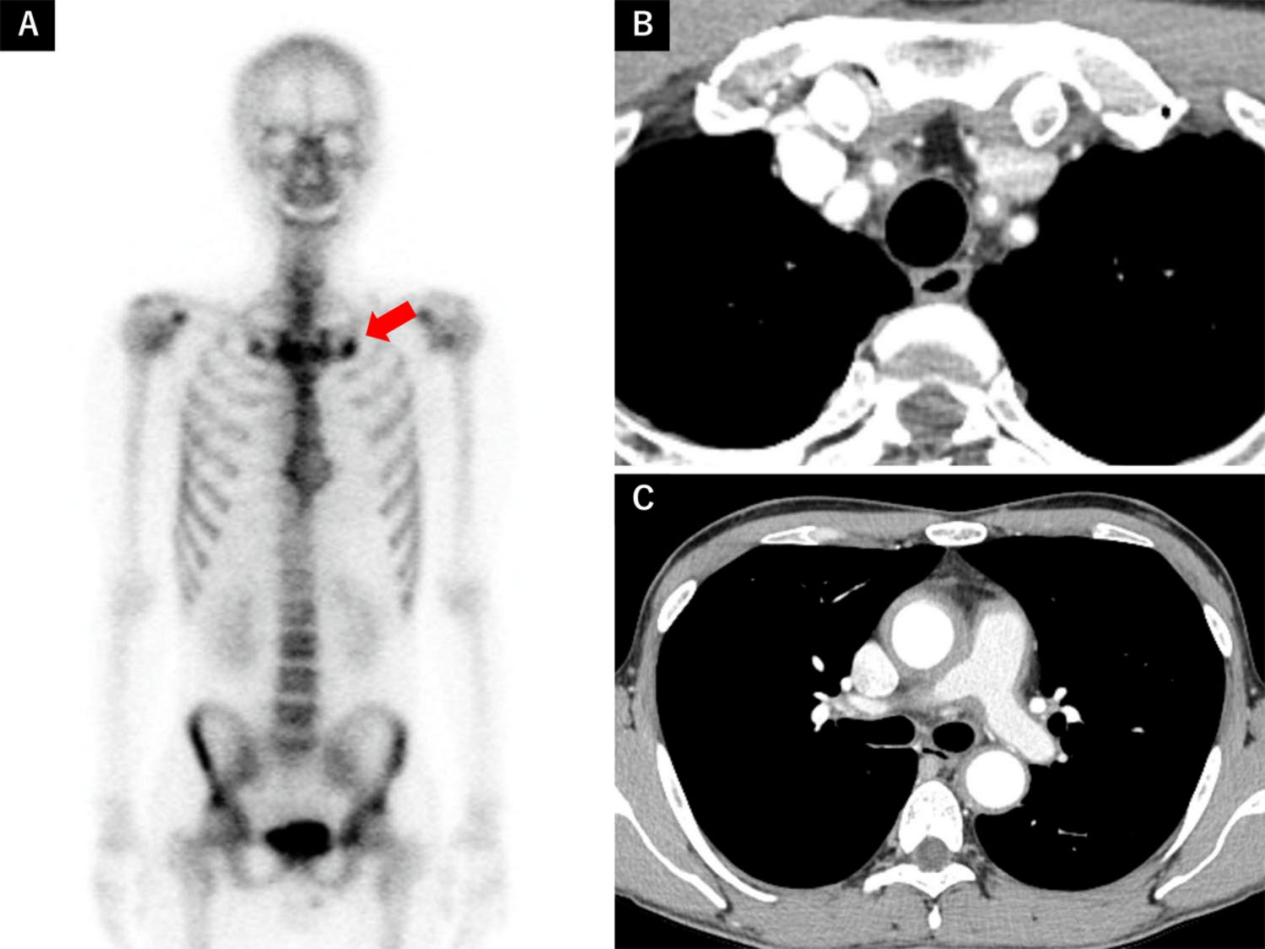
**A** 99 m-Tc bone scintigraphy showing tracer accumulation in the first sternal rib joint. **B C** Chest CT demonstrating circumferential wall thickening with a contrast effect of the ascending to descending aorta, bilateral common carotid arteries, the left subclavian artery, and the main trunk of the pulmonary artery

A 31-year-old man was referred to our hospital with anterior chest pain that had persisted for one month. Physical examination revealed swelling of the right sternoclavicular joint and diffuse tenderness in the anterior thoracic region. No acne-like skin lesions or pustules were present on the palms or soles. His body temperature was 36.4 °C, and there was no difference in pulse or blood pressure between his arms. Laboratory examinations showed a markedly elevated CRP level (14.62 mg/dL), while ANA, RF, and ACPA were negative. Plain CT of the chest revealed mild bone hyperplasia around both first sternocostal joints. Given the suspicion of SAPHO syndrome, 99 m-Tc scintigraphy was performed, demonstrating tracer accumulation suggestive of osteomyelitis in the same region (Fig. 2A). SAPHO syndrome was diagnosed based on Benhamou’s criteria; however, the markedly elevated inflammatory response prompted further investigation. A bruit was detected in his neck upon further examination, and a contrast-enhanced CT revealed arterial wall thickening from the ascending to descending aorta, as well as moderate stenosis of the bilateral common carotid arteries and mild stenosis of the left subclavian artery (Fig. 2B, C). No collaterals were observed. Based on the 2022 ACR/EULAR classification criteria, the patient was diagnosed with TAK coexisting with SAPHO syndrome. His anterior chest pain improved rapidly after initiating PSL (50 mg/day); however, CRP levels did not normalize. Consequently, methotrexate (10 mg/week) and tocilizumab (162 mg/week) were introduced, leading to complete symptom resolution. Over the next 10 months, PSL was tapered to 10 mg/day without relapse.

## Literature review

A systematic literature search was conducted in August 2024 using the PubMed and Web of Science databases. Given that SAPHO syndrome encompasses a spectrum of heterogeneous diseases with osteoarticular manifestations, as noted in the Introduction, the search included the following keywords: (“Takayasu arteritis” OR “Takayasu’s arteritis”) AND (“synovitis, acne, pustulosis, hyperostosis, and osteitis syndrome” OR “SAPHO syndrome” OR “chronic recurrent multifocal osteomyelitis” OR “CRMO” OR “acquired hyperostosis syndrome” OR “AHS” OR “pustulotic arthro-osteitis” OR “PAO” OR “osteomyelitis”). References from the retrieved articles were also screened for additional relevant studies. Data on patients with SAPHO syndrome complicated by TAK were extracted from articles published between 1995 and 2023. The exclusion criteria were: (1) cases in which the clinical course of the patient was not reported and (2) cases of osteomyelitis that could not be classified as SAPHO syndrome according to established diagnostic criteria [2, 3]. Two authors (SS and DK) independently reviewed all articles, including titles, abstracts, and full texts. The following parameters were extracted: clinical manifestations, temporal sequence and diagnostic interval of both diseases, anatomical distribution of osteoarticular and vascular lesions, and treatment approaches.

## Results

A literature search identified 11 patients with TAK complicated by SAPHO syndrome [13–22]. Patient characteristics are summarized in Table 1. The mean age at diagnosis was 24.5 (range: 4–49) years, and 9 patients (81.8%) were female. SAPHO syndrome was diagnosed before TAK in seven cases (63.6%), while TAK preceded SAPHO syndrome in two cases (18.2%). In the remaining two cases (18.2%), both conditions were diagnosed simultaneously. The interval between diagnoses ranged from 1 month to 12 years. Among the 10 patients with available treatment data, nine received glucocorticoids, six received immunosuppressants, three were treated with biological agents, and one received a JAK inhibitor. There was no clear association between the anatomical distribution of osteoarticular and vascular lesions.

**Table 1:** Clinical characteristics of Takayasu arteritis complicated by SAPHO syndrome in the literature

Age	Sex	Prior diagnosis	Diagnostic interval between SAPHO and TAK	Osteo-articular involvement	Vascular lesion due to angiographic classification*	Treatment	Ref
4	M	SAPHO	3 years	ACW, Tibia, Wrist, Ankle	2 A	PSL, Colchicine, AZP Surgery	13
21	F	TAK	7 years	ACW, Scapula	5	N.D	14
35	F	SAPHO + TAK	Simultaneous	Ulna, Radius, Tibia	1	PSL	15
49	F	SAPHO + TAK	Simultaneous	ACW	2 A	PSL, Surgery	16
10	F	SAPHO	2 years	Right mandible	5	PSL, MMF Endovascular therapy	17
27	F	SAPHO	8 months	Mandible, ACW	1	PSL	18
20	F	SAPHO	12 years	Right limb	N.D	PSL, TAC, TCZ	18
15	F	SAPHO	10 years	Femur, Tibia, Sacrum	3	PSL, MTX, IFX	19
41	F	SAPHO	2 years	ACW	2 A	PSL, CPA, CyA, TCZ, GLM	20
12	F	TAK	10 months	Skull	1	PSL	21
36	M	SAPHO	1 month	ACW	1	SASP, MTX, TOFA	22
27	F	SAPHO	5 years	ACW	2B	PSL, IFX, UST	Case 1
31	M	SAPHO + TAK	Simultaneous	ACW	2B	PSL, MTX, TCZ	Case 2

SAPHO, SAPHO syndrome; TAK, Takayasu arteritis; ACW, anterior chest wall; PSL, prednisolone; AZP, azathioprine; MMF, mycophenolate mofetil; TAC, tacrolimus; MTX, methotrexate; CPA, cyclophosphamide; CyA, cyclosporin A; SASP, salazosulphapyridine; TCZ, tocilizumab; IFX, infliximab; GLM, golimumab; TOFA, tofacitinib; UST, ustekinumab; Ref, reference; N.D., not described

^*^ Vascular lesions according to angiographic classification[23]

## Discussion

We present two patients with TAK complicated by SAPHO syndrome, both of whom initially presented with anterior chest pain and were subsequently diagnosed with TAK. Their symptoms improved with treatment using glucocorticoids and biological agents. In addition, we reviewed similar cases reported in the literature.

Our patients presented with anterior chest pain as the chief complaint, which is the most common symptom of SAPHO syndrome. Physical examination revealed sternoclavicular arthritis. However, widespread tenderness in the anterior thoracic region and an inadequate response to NSAIDs raised suspicion of an additional underlying condition. It has been reported that 15.9% of patients with TAK experience body pain, including chest, back, or abdominal pain, as their primary symptom at disease onset [8]. Inflammation of the aorta itself can cause neck and trunk pain due to the distribution of nerves in the adventitial-medial boundary of most blood vessels [24]. The mechanism of aortic pain in aortitis may involve both mechanical factors, such as reduced elastic properties and compliance of the aortic wall, and chemical factors, including the release of inflammatory cytokines [25, 26]. Both of our patients had aortitis in the thoracic region, affecting the ascending aorta, aortic arch, and descending aorta; therefore, their anterior chest pain was attributed to both SAPHO syndrome-associated arthritis and TAK-related vascular inflammation. Our literature review indicates that 63.6% of cases involving coexisting SAPHO syndrome and TAK had SAPHO syndrome as the initial diagnosis. As mentioned above, patients with TAK may present with anterior chest pain; however, this condition may be overlooked in patients already diagnosed with SAPHO syndrome. Although laboratory findings in SAPHO syndrome are often nonspecific, inflammatory markers are generally only mildly elevated [27, 28]. A disproportionately high level of inflammation relative to the severity of arthritis or osteitis should prompt consideration of a comorbid TAK diagnosis.

TAK is characterized by both autoimmune and autoinflammatory processes and is known to be associated with various inflammatory diseases [29]. This study suggests that SAPHO syndrome may be one of the conditions that can coexist with TAK. A positive association between TAK and SpA, a disease closely related to SAPHO syndrome, has been previously reported. An observational retrospective study demonstrated that 8.8% of patients with TAK had a form of SpA spectrum disease [30]. In this study, patients with TAK-SpA exhibited an earlier onset of vascular symptoms compared to those without SpA. Furthermore, consistent with our findings, SpA-related symptoms frequently preceded TAK symptoms. Although the precise mechanisms underlying the association between TAK and SAPHO syndrome remain unclear, they likely share genetic and immunopathogenic factors. Various inflammatory cytokines play a pivotal role in both diseases, particularly those related to Th1 and Th17 pathways, including IL-6, IL-12, IL-17, IL-22, IL-23, IFN-γ, and TNF-α [31, 32]. Neutrophilic infiltration of vascular lesions has been observed in patients with TAK, with Th17 cells facilitating neutrophil recruitment through IL-17 A secretion [33, 34]. On the other hand, SAPHO syndrome has been associated with an imbalance between Th17 cells and Treg cells, which may be driven by a reduction in NK cells [35]. The depletion of NK cells in SAPHO syndrome has been postulated to result from their recruitment to inflammatory target tissues, a phenomenon also observed in TAK. Notably, NK cell accumulation in the aortic media distinguishes TAK from GCA, another form of large-vessel vasculitis [36]. In addition, recent studies have reported that gut dysbiosis is linked to both the development and severity of TAK and SAPHO syndrome [37, 38]. These findings suggest that the two diseases may share common pathogenic mechanisms, and their coexistence may be underrecognized.

There is no established treatment strategy for patients with TAK complicated by SAPHO syndrome. However, various biological agents have been used in refractory cases, as demonstrated in Table 1. Current TAK management guidelines recommend TNF-α inhibitors and tocilizumab for refractory cases [12, 39], with additional options including rituximab, IL-12/23 inhibitors, and JAK inhibitors [40]. For SAPHO syndrome, TNF-α inhibitors have been reported as the most effective biologics agents, while IL-1 and IL-17/IL-23 inhibitors are considered second-line treatments [41]. In our cases, different biological agents were administered: infliximab and ustekinumab in Case 1, and tocilizumab in Case 2. In Case 1, the disease activity of both TAK and SAPHO syndrome was similarly elevated, prompting the initial selection of infliximab, which successfully controlled both aortitis and SAPHO-related symptoms. After discontinuing infliximab, disease activity remained stable with ustekinumab, a monoclonal antibody targeting IL-12/23p40. It has been reported that IL12B is associated with the pathophysiology of TAK and that plasma levels of IL-12p40 are elevated in patients with TAK [42, 43]. Although clinical evidence is limited to reports from a small number of cases, a good clinical response and safety have been demonstrated [44]. In contrast, in Case 2, while anterior chest pain and swelling of the sternoclavicular joint improved rapidly with glucocorticoid treatment, inflammatory activity persisted, suggesting that this inflammation was primarily due to TAK. Considering that tocilizumab is currently the only biologic approved for TAK in Japan, we selected it as the initial biologic therapy, which led to symptom resolution. Although switching to TNF-α inhibitors will be considered if symptoms flare up, these cases highlight the importance of tailoring treatment strategies based on the predominant disease activity.

In conclusion, we present two cases of TAK complicated by SAPHO syndrome and review similar cases from the literature. Since SAPHO syndrome frequently precedes TAK, and the onset of TAK may be delayed by several years, clinicians should remain vigilant for signs of vascular involvement in patients with SAPHO syndrome who exhibit unexplained clinical signs. While our findings suggest a potential immunopathogenic link between the two diseases, further research involving larger patient cohorts is needed to clarify their relationship and optimize treatment strategies.

## Data Availability

Not applicable.
